# Relationship of Internalized Transnegativity and Protective Factors With Depression, Anxiety, Non-suicidal Self-Injury and Suicidal Tendency in Trans Populations: A Systematic Review

**DOI:** 10.3389/fpsyt.2021.636513

**Published:** 2021-05-20

**Authors:** Marc Inderbinen, Kristin Schaefer, Andres Schneeberger, Jens Gaab, David Garcia Nuñez

**Affiliations:** ^1^Division of Clinical Psychology and Psychotherapy, Faculty of Psychology, University of Basel, Basel, Switzerland; ^2^Department of Plastic, Reconstructive and Aesthetic Surgery and Hand Surgery, Basel University Hospital, Basel, Switzerland; ^3^Psychiatric Services Graubünden, Chur, Switzerland; ^4^Center for Gender Variance, Basel University Hospital, University of Basel, Basel, Switzerland

**Keywords:** transgender (LGBT), mental health, gender minority stress, internalized transphobia, internalized transnegativity

## Abstract

Discrimination heavily impacts the lives of trans populations and causes adverse mental health outcomes. As stated by the Gender Minority Stress Model self-stigmatization could play an important role in this process. The aim of this systematic review is to investigate whether there is a positive association between self-stigmatization and mental health and to identify mediation factors. Studies which quantitatively investigated the association between internalized transnegativity and selected mental health outcomes (depression, anxiety, non-suicidal self-injury, suicidal tendency) in self-identified trans populations were included. Comprehensive search of 5 large databases in June 2020 and the following screening and selection procedure, performed by two researchers separately, identified 14 studies which met criteria. The relationship to be studied was reported with correlation and/or mediation analysis of cross-sectional data. IT was directly positively associated with depression, anxiety and suicidal tendency in most of the reviewed studies. Data indicates links between self-stigmatization and other general mental health stressors such as rumination and thwarted belongingness. Community connectedness showed to be the strongest protective factor for mental health impairments. These results should be considered in transition counseling. More research is needed to better understand the underlying mechanisms of the GMSM and to address unsolved operationalization and measurement issues.

## Introduction

Trans^1^ populations face pervasive discrimination, violence and rejection in virtually all cultures and areas (2–4). More precisely, gender identity minorities report less peer and family support than sexual minorities,^2^ which may also contribute to their higher vulnerability for mental health impairments (6). It is reported that trans populations in the U.S. have an increased lifetime prevalence of depression (range: 48–62%) (7, 8) and anxiety (range: 26–38%) (9) when compared to the general population (depression: 16.6%, combined anxiety disorders: 28.8%) (10). With a range from 28 to 40%, the prevalence of suicidal ideation (SI) and suicide attempts (SA) among trans individuals is extremely high (11). It is significantly increased compared to cis people with 5–11% (12, 13). The vulnerability to non-suicidal self-injury (NSSI) thoughts and behaviors in trans people is seen in both, people in early coming-out stages (13) and post-transitioned people (14). In this article, we refer to SA and SI as suicidal tendency (15).

### Internalized Transnegativity and Its Consequences

In order to link stigmatization with adverse mental health in trans populations, Hendricks and Testa (16, 17) formulated the Gender Minority Stress Model (GMSM) for trans and gender-nonconforming people based on Meyer's Sexual Minority Stress Hypothesis (SMSH) (18, 19). The GMSM describes that as a social minority, trans persons are exposed to socially conditioned and structurally enforced stress factors, also called “distal stressors” (i.e., discrimination, rejection and victimization, non-recognition of gender). By constantly experiencing these distal stressors, many trans people begin to internalize the negative beliefs of society about themselves, which can lead to the development of negative expectations about their future, the concealment of their real gender identity and the development of “internalized transnegativity” (IT). In the existing literature, different conceptualizations of IT are present. Hughto et al. (20) presented a conceptual framework of IT which focused on interrelations with structural- (e.g., societal norms), interpersonal- (e.g., everyday interactions), and individual stigma (concealment, avoidance, internalized stigma). However, more recently a more comprehensive definition of IT integrating emotional processes has been conceptualized (21). According to this definition, IT consist of four inter-related dimensions: (2) pride in trans identity, (3) investment in passing as cis gender person, (4) alienation from other trans people, (6) shame.

Over the last few years, research has provided multiple clues to the plausibility of the SMSH. Thus, several studies have demonstrated the direct negative impact of distal factors on the mental health of lesbian, gay and bisexual (LGB) (22). The research in LGB populations also shows, that there is most likely a positive association between internalized stigma and mental health (23). It seems that of all the proximal stressors that affect LGB persons, internalized stigma has the strongest association with SI (24), depression (25) and social anxiety (26). Focusing on resilience the SMSH includes factors such as connectedness to the community and pride in belonging to a minority. Dependencies between internalizing stigma and protective factors in LGB populations have been discussed (27). It is reported that openness about one's sexual orientation (28) and social support (29) served as protective factors for mental health issues in LGB persons. Further Postuvan et al. (30) reported in a systematic review that a lack of community connectedness contributed to the development of suicidal behavior (i.e., SI, SA and complete suicide) in LGB and trans people.

However, these results from sexual minorities cannot be directly applied to gender minorities. Although both groups are subject to the same stress mechanisms there are also specific stressors. Thus, there is still a lack of research assessing minority stressors as well as protective factors and their relationship with mental health outcomes in trans populations. While there is a growing body of knowledge that confirms the direct impact of gender related discrimination, rejection, victimization and non-affirmation of gender identity on mental health of trans persons (8, 31, 32), there exists, according to our knowledge, no systematic review focusing on the relationship between proximal minority stressors, protective factors and the mental health of this population. Considering that it has been postulated that as a minority stress process, IT negatively affects health outcomes among trans people (33), this systematic summary work within the GMSM framework is of crucial importance.

### Objectives

The purpose of this systematic review was to examine the model's suggested association between proximal minority stressors, protective factors and adverse mental health outcomes as well as its implications for clinical work and research. Given that literature on the LGB population considers internalized stigma as the major factor among proximal stressors, we are focusing on IT in this review. As already suggested by other authors, we include the terms “internalized transphobia” and “self-stigma” under this term. Accordingly, two research question to guide this review were defined:

- Is IT positively associated with suicidal tendency, NSSI, depression and/or anxiety in trans populations?
- Are pride and community connectedness protective factors for suicidal tendency, NSSI, depression and/or anxiety in trans populations?

## Methods

### Search Strategy

The search strategy was structured following the Preferred Reporting Items for Systematic reviews and Meta-Analysis (PRISMA) Statement (34). Existing literature was searched in five electronic databases (Pubmed, Web of Science, PsycInfo, Embase and CINAHL). Where possible, we used medical subject heading (MeSH) terms. Otherwise, studies were selected based on titles and abstracts. The keywords were sorted by Participants, Interventions and Comparison and Outcome (PICO) (Table 1) (35). Database-specific search-strategies can be found in the Appendix. The search was performed by two authors (MI and KS) in June 2020. After extensive research of databases gray literature research (e.g., google scholar, search of references of included papers) was performed. Since the included studies were approved by the corresponding ethics committees, this review was exempt from institutional review board.

**Table 1:** PICO keywords.

P(ATIENTS)	trans* OR transsex* OR trans OR gender dysphor* OR gender minorit* OR LGBT OR gender identit* OR male-to-female OR female-to-male OR trans men OR trans women
I(NTERVENTION)	transphobia OR stigma* OR minority stress OR sexual?stigma OR transnegativ* OR cissexism OR discrimination
C(OMPARISON)	internali?ed transphobia OR internali?ed stigma OR self?stigma OR internali?ed cissexism OR internali?ed transnegativ*
O(UTCOME)	suicide risk OR suicide attempts OR suicide OR suicidal ideation OR suicide ideation OR suicidal behavior OR suicidality OR self-harm behavior OR self-harm OR non?suicidal self-harm OR self-injury behavior OR self-injury OR non?suicidal self-injury OR depress* OR anxiety OR affective disorder OR mood disorder OR anxiety disorder

### Selection Procedure

**Figure 1:**
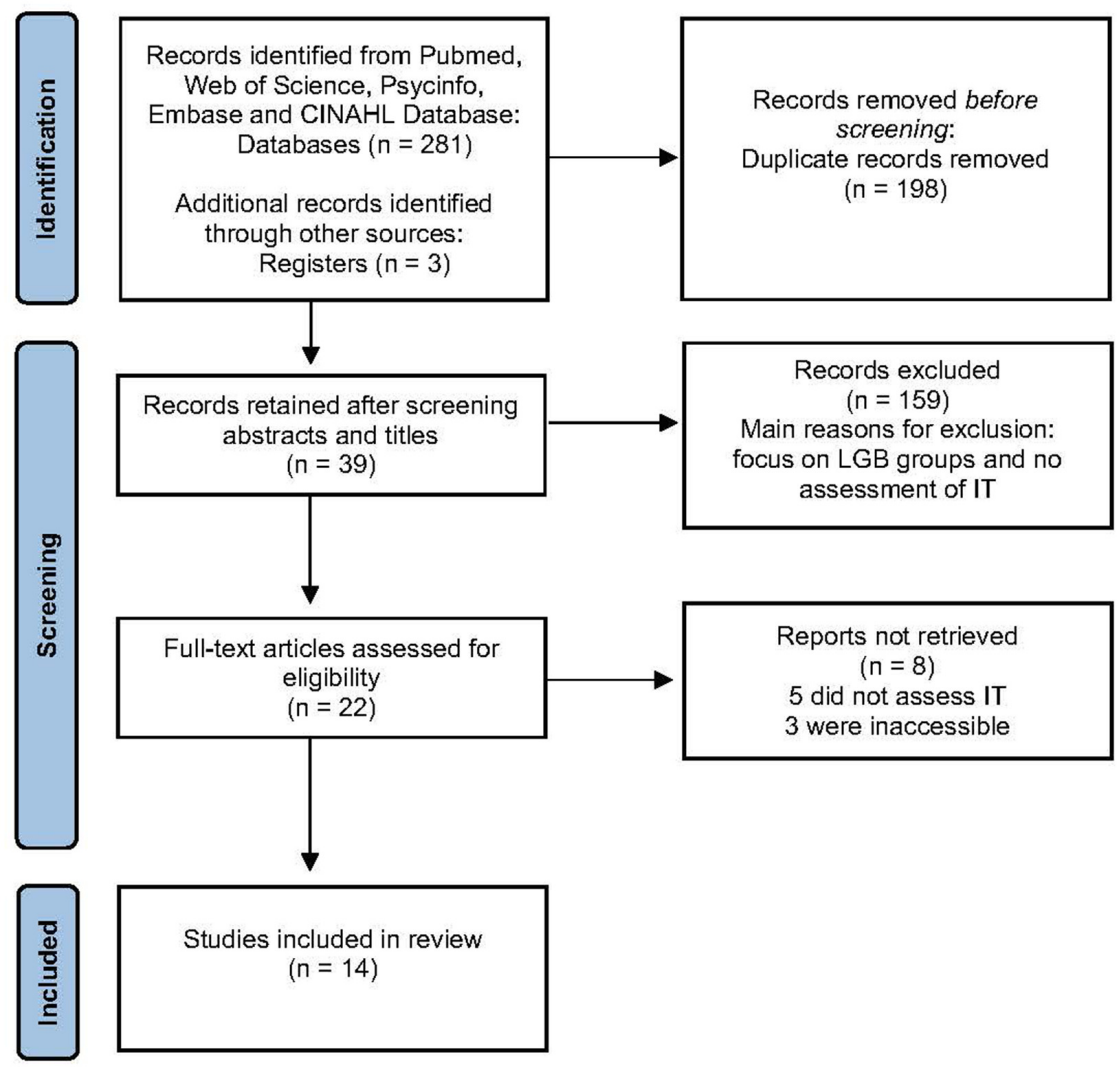
PRISMA flow-diagram of systematic search.

All references were stored in a correspondence database program (EndNote X8). Two Authors (MI and KS) independently reviewed the results and sorted articles out from the final selection according to predetermined criteria (Tables 2, 3). Non-English, non-German, non-human reviews, books, opinion pieces, and case reports were excluded. Since terms such as “gender non-conforming” or “genderqueer” are vague and occasionally adopted by cis people who are hardly exposed to the same exclusion dynamics as the trans community, we decided to include solely studies providing clear definition of participants' “trans” identification labels in their sample. Considering that “internalized transphobia,” “internalized transnegativity,” “internalized cissexism” and “self-stigma in trans persons” are used complementary and overlapping in the literature, no exclusion criteria have been formulated regarding these terms. Studies working with an identical sample were only included into the review if secondary analysis revealed additional results. Any disagreement over the eligibility of particular studies was resolved through discussion with another reviewer (DGN). Figure 1 shows the summary of search strategy and the selection procedure.

**Table 2:** Exclusion criteria.

Trans Populations	Exclude: studies providing a vague definition of their trans sample including only cis LGB persons, no differentiation between the sexual (LGB) and gender (T) minorities
Assessment of mental health indicators	Exclude: studies providing exclusively data on substance consume, somatization, personality disorders
Assessment of IT	Exclude: studies providing exclusively data on internalized homophobia, distal minority stressors, enacted stigma, no differentiation between proximal and distal stress factors

**Table 3:** Inclusion criteria.

Type of article	Empirical article including original data
Age of the sample	Adults (over the age of 18)
Study participants	Trans individuals in a broader sense, which also includes “transsexuals,” “transgenders,” “people with gender dysphoria,” “people with gender identity disorder,” “LGBT,” “male-to-female,” “female-to-male,” “trans men” and “trans women”
Measure of comparison	IT
Assessment of outcome	NSSI or SI or SA or depression or anxiety

*NSSI, non-suicidal self- injury, SI, suicidal ideation; SA, suicide attempts*.

### Data Extraction and Analysis

The following information was extracted from each included study: information on the country of origin, the year of publication, sample characteristics (sample size, and subgroup of participants), level of evidence (Table 4) (49) types of IT measurements and types of NSSI, SI, SA, depression and/or anxiety assessments and mention of possible protective factors (Tables 5–**7**). Also, the findings of the studies regarding the relationships between IT and mental health as well as suicidal tendency and protective factors were summarized (**Tables 8**, **9**). In case of missing data, the authors were contacted and, if necessary, data in this analysis were supplemented.

**Table 4:** Article overview and demographics as reported in studies.

	**References**	**Country of origin**	**Self-identification**	**Participants**	**Sample size**	**Age (mean ± SD in years)**	**Level of evidence**
Resulting from systematic literature research	Fredriksen-Goldsen et al. (36)	USA	LGBT	174 (7%) trans 2,372 (93%) non-trans	*N* = 2,560	Total: 66.47 ± 9.08 Trans: 60.97 ± 7.96 Non-trans: 66.87 ± 9.03	4
	Wilson (37)	USA	Trans or cis gender	43 (5.8%) trans men 135 (18.2%) trans women 111 (14.9%) cis men 290 (39.0%) cis women 121 (16.3%) genderqueer/androgynous 43 (5.8%) other	*N* = 743	Total: 35.00 ± 10.84	4
	Perez-Brumer et al. (38)	USA	Transgender	532 (43.29%) trans men 697 (56.71%) trans women	*N* = 1,229	Total: 32.74 ± 11.96	4
	Testa et al. (17)	USA	Transgender identity	251 (29.8%) trans men 138 (16.4%) trans women 97 (11.5%) women 251 (29.8%) men 184 (21.9%) gender-queer 9 (1.1%) cross-dresser	*N* = 844	Trans women: 36.81 ± 14.54 Trans men: 31.19 ± 11.46 Cis Women: 36.81 ± 14.54 Cis Men: 31.47 ± 10.61 Gender-queer: 27.26 ± 10.11 Cross-dresser: 40.50 ± 17.00	4
	Marshall et al. (39)	Argentina	Trans men or trans women	44 (9.1%) trans men 438 (90.9%) trans women	*N* = 482	Total Median: 30.00 Interquartile range (IQR): 25–37	4
	Tebbe and Moradi (40)	USA	Trans identity	90 (26.9%) trans men 110 (32.8%) trans women 129 (38.2%) gender-non-conforming	*N* = 329	Total: 25.21 ± 6.58	4
	Hoy-Ellis and Fredriksen-Goldsen (41)	USA	Transgender	50 (35.97%) trans men 89 (64.03%) trans women	*N* = 174	Total: 60.97 ± 7.96	4
	Testa et al. (42)	USA	Transgender	372 (45.6%) trans men 252 (30.9%) trans women 148 (18.1%) female to different gender 37 (4.5%) male to different gender 7 (0.9%) intersex	*N* = 816	Total: 32.53 ± 13.13	4
	Timmins et al. (43)	UK	Transgender	346 (28.6%) trans men 377 (31.2%) trans women 484 (47.1%) non-binary	*N* = 1,207	Total: 28.50	4
	Staples et al. (44)	USA	Transgender	132 (55.9%) other 58 (24.6%) trans men 24 (10.2%) trans women 22 (9.3%) non-binary	*N* = 237	Total: 28.00 ± 6.90	4
	Sapareto (45)	USA	Transgender	Gender assigned at birth: 12 (41.38%) male 17 (58.62%) female	*N* = 29	Not reported	4
Resulting from additional sources	Brennan et al. (46)	USA	Individuals who identify as a gender different than the sex assigned to them at birth	24 (29%) trans men 33 (40%) trans women 26 (31%) other gender-non-conforming identity	*N* = 83	Range: 19–70	4
	Jäggi et al. (47)	CH	Trans identity	43 (30%) trans masculine 75 (52%) trans feminine 26 (18%) non binary	*N* = 143	Trans feminine: 51.5 ± 17.1 Trans masculine: 36.0 ± 12.8 Non-binary: 42.2 ± 24.4	4
	Scandurra et al. (48)	IT	Transgender identity	75 (50.3%) transgender women 74 (49.7%) transgender men	*N* = 149	Transgender women: 37.2 ± 12.2 Transgender men: 29.2 ± 7.8	

**Table 5:** Description of studies' instruments to assess mental health.

**References**	**Suicide attempts and self-harm**	**Anxiety**	**Depression**
Fredriksen-Goldsen et al. (36)			Short form of the Center for Epidemiological Studies Depression Scale (CES-D)
Wilson (37)	Self-Harm Questionnaire	Depression Anxiety and Stress Scale (DASS)	Depression Anxiety and Stress Scale (DASS)
Perez-Brumer et al. (38)	2-item questioning lifetime SA and past 12-months SA		
Testa et al. (17)		Shortened version of the Social Phobia Inventory (Mini-SPIN)	Center for Epidemiological Studies Depression Scale (CES-D)
Marshall et al. (39)	1-item questioning about lifetime SA		
Tebbe and Moradi (40)	Suicidal Behaviors Questionnaire–Revised (SBQ-R)		Center for Epidemiological Studies Depression Scale (CES-D)
Brennan et al. (46)	1-item questioning about lifetime SA	Beck Anxiety Inventory (BAI)	Center for Epidemiological Studies Depression scale (CES-D)
Hoy-Ellis and Fredriksen-Goldsen (41)			Short form of the Center for Epidemiological Studies Depression Scale (CES-D)
Testa et al. (42)	Suicidal Ideation Scale (SIS)		
Timmins et al. (43)		Generalized Anxiety Disorder Scale (GAD-7)	Patient Health Questionnaire (PHQ-9)
Jäggi et al. (47)			Allgemeine Depressionsskala (ADS-K), german equivalent to (CES-D)
Staples et al. (44)	Beck Scale for Suicidal Ideation (BSI), Deliberate Self-Harm Inventory (DSHI)		
Sapareto (45)	Suicide Behaviors Questionnaire-Revised (SBQ-R)	Zung Self-Rating Anxiety Scale (SAS)	Goldberg Depression Scale (GDS)
Scandurra et al. (48)		Beck Anxiety Inventory (BAI)	Center for Epidemiological Studies Depression scale (CES-D)

## Results

### Study Characteristics

Our search identified 14 studies (Table 4) which met our criteria (see Figure 1), including a total of 4.913 trans persons (17, 36–40, 42–45, 47–50). In almost all cases, participants' inclusion was assessed through self-identification as “transgender,” “trans men” or “trans women” (17, 37–40, 42–46, 48, 50). While seven studies included mostly trans women (37, 38, 40, 45–48, 50), four included more trans men (17, 42, 44) and in one study most participants identified as non-binary [Transgender participants were considered non-binary if they reported a gender identity that was anything other than exclusively male or female, (40, p. 339)]. Regarding socio-demographic sample characteristics the studies' samples were predominantly Caucasian (17, 37–40, 42–48).

The studies included in this review assessed depression (17, 29, 37–40, 43, 45, 46, 48), anxiety (17, 37, 43, 45, 46, 48) suicidal tendency (38–40, 42, 44–46) and NSSI (37, 44) with different instruments (Table 5). Furthermore, various approaches to assess IT (Table 6) and protective factors (Table 7) were used.

**Table 6:** Description of studies' instruments to assess mental health.

**References**	**Suicide attempts and self-harm**	**Anxiety**	**Depression**
Fredriksen-Goldsen et al. (36)			Short form of the Center for Epidemiological Studies Depression Scale (CES-D)
Wilson (37)	Self-Harm Questionnaire	Depression Anxiety and Stress Scale (DASS)	Depression Anxiety and Stress Scale (DASS)
Perez-Brumer et al. (38)	2-item questioning lifetime SA and past 12-months SA		
Testa et al. (17)		Shortened version of the Social Phobia Inventory (Mini-SPIN)	Center for Epidemiological Studies Depression Scale (CES-D)
Marshall et al. (39)	1-item questioning about lifetime SA		
Tebbe and Moradi (40)	Suicidal Behaviors Questionnaire–Revised (SBQ-R)		Center for Epidemiological Studies Depression Scale (CES-D)
Brennan et al. (46)	1-item questioning about lifetime SA	Beck Anxiety Inventory (BAI)	Center for Epidemiological Studies Depression scale (CES-D)
Hoy-Ellis and Fredriksen-Goldsen (41)			Short form of the Center for Epidemiological Studies Depression Scale (CES-D)
Testa et al. (42)	Suicidal Ideation Scale (SIS)		
Timmins et al. (43)		Generalized Anxiety Disorder Scale (GAD-7)	Patient Health Questionnaire (PHQ-9)
Jäggi et al. (47)			Allgemeine Depressionsskala (ADS-K), german equivalent to (CES-D)
Staples et al. (44)	Beck Scale for Suicidal Ideation (BSI), Deliberate Self-Harm Inventory (DSHI)		
Sapareto (45)	Suicide Behaviors Questionnaire-Revised (SBQ-R)	Zung Self-Rating Anxiety Scale (SAS)	Goldberg Depression Scale (GDS)
Scandurra et al. (48)		Beck Anxiety Inventory (BAI)	Center for Epidemiological Studies Depression scale (CES-D)

**Table 7:** Description of studies' instruments to assess protective factors.

**References**	**Instrument**	**Measured construct**	**Description**	**Cronbach's alpha of the study population**	**Number of items**	**Item example**
Fredriksen-Goldsen et al. (36)	Adapted Social Support Instrument	a) Social support b) Positive feeling of community belonging		a) 0.85	a) 4 b) 1	
Testa et al. (17)	Interpersonal Needs Questionnaire (INQ-12) Trans Identity Survey (TGIS)	a) Community connectedness b) Pride		a) 0.88		
Tebbe and Moradi (40)	Family, Friend, and Significant Other subscales of the Multidimensional Scale of Perceived Social Support (MSPSS)	Friend support	Friends subscales	0.91	4	
Brennan et al. (46)	Gender Minority Stress and Resilience measure (GMSR)	a) Community connectedness b) Pride	GMSR subscale resilience factors (community connectedness and pride)	a) 0.89 b) 0.82	a) + b) 13	“I feel part of a community of people who share my gender identity.”
Jäggi et al. (47)	Gender Minority Stress and Resilience measure (GMSR)	Community connectedness	GMSR subscale community connectedness		5	“I feel connected to other people who share my gender identity.”
Scandurra et al. (48)	Resilience Scale (RS)	Resilience	Resilience was conceptualized as a personal characteristic buffering the negative effects of stress and promoting adjustment	.90	10	“When I'm in a difficult situation, I can find my way ot of it.”

Few studies have investigated whether there were gender differences in reported depression and suicidal tendency. One study reported that trans feminine identity was associated with decreased odds of lifetime suicide attempts (38). Other studies, however, reported no differences in the frequency of SI (42), depression (47), SI and SA (40). Gender differences in anxiety and NSSI were not investigated in the included studies. Also, hardly any gender differences in reported IT were investigated. Jäggi et al. (47) reported no significant differences between IT scores in trans feminine and trans masculine persons. One study showed that age had an influence on depression and anxiety symptoms (46). As age increased, there was a significant decrease in symptoms of depression and anxiety (46). In the reviewed studies, no gender differences or age differences in correlation and mediation analyses were examined and reported.

### Relationships Between IT, Depression and, Anxiety

Drawing from the results of most studies (17, 36, 37, 40, 41, 43, 46, 47) the positive association between IT and depression seems to be consistent: trans people with more IT reported more depression (Table 8). Only in a study with a small sample (*N* = 29) this relation did not reach a significant level (45). Furthermore, IT was even found to be a strong significant risk factor for physical and mental health impairments in an elderly sample (age > 50 years), indicating the applicably of the model across different age groups (41). One study focused on shame and alienation as components of IT, and reported for both direct positive associations with depressive symptomology (48).

**Table 8:** Results overview, internalized transnegativity.

**Study**	**Outcome**	**Relationships of internalized transnegativity**	
		**Direct effects**	**Indirect effects**
Fredriksen-Goldsen et al. (36)	1) Depressive symptomology	a) B = 2.20^***^	IT as mediator 1) Path coefficient = 0.034^***^
Wilson (37)	1) Depressive symptomology 2) Anxiety	a) b = 0.33^**^ b) b = 0.29^**^	
Testa et al. (17)	1) Depressive symptomology 2) Anxiety symptomology	a) B = 0.49^*^ b) B = 0.38^*^	
Perez-Brumer et al. (38)	1) Lifetime SA	a) AOR = 1.18^*^, 95% CI (1.04–1.33)	
Marshall et al. (39)	1) Lifetime SA	a) AOR = 2.06^***^	
Tebbe and Moradi (40)	1) Depressive symptomology 2) Suicide risk	a) b = 0.21^**^ b) b = 0.29^**^	IT via depression 2) B = 0.019, 95% CI (0.003–0.039)
Brennan et al. (46)	1) Depressive symptomology	IT, negative expectations and non-disclosure combined a) b = 0.270^*^	
Hoy-Ellis and Fredriksen-Goldsen (41)	2) Depressive symptomology	a) b = 1.90	IT via perceived general stress 1) b = 5.93^***^
Testa et al. (42)	1) SI 2) Thwarted belongingness 3) Perceived burdensomeness	a) b = 0.41^***^ b) b = 0.43^***^ c) b = 0.49^***^	IT via thwarted belongingness 1) B = 0.03, 95% CI (0.02, 0.05) via perceived burdensomeness 1) B = 0.18, 95% CI (0.14, 0.22)
Timmins et al. (43)	1) Depressive symptomology 2) Anxiety symptomology 3) Well-being	a) b = 0.57^***^ b) b = 0.32^***^	Prejudice events via IT to combined1) + 2)+ 3) b = 0.11^***^ IT via rumination to combined 1) + 2) + 3) b = 0.13^***^
Jäggi et al. (47)	1) Depressive symptomology	a) r = 0.42^**^	IT, negative expectations for future events and nondisclosure of identity combined 1) β = 2.47^*^
Staples et al. (44)	1) SI 2) NSSI	a) r = 0.36^***^ b) r = 0.06	IT as mediator 1) B = 0.07, 95% CI = (0.02, 0.13)^**^ 2) B = 0.01, 95% CI = (−0.04, 0.06)
Sapareto (45)	1) Depressive symptomology 2) Anxiety symptomology 3) SI	a) B = 0.01 b) B = 0.90 c) B = −1.34^*^	
Scandurra et al. (48)	1) Depressive symptomology 2) Anxiety symptomology	a) Shame r = 0.43^***^, alienation r = 0.38^***^ b) Shame r = 0.32^***^, alienation r = 0.33^***^	Shame and/or alienation as mediator 1) Shame b = 0.55, 95% CI (0.08, 1.55)^*^ Alienation b = 0.60, 95% CI (0.08, 1.55)^*^ 2) Alienation b = 0.91, 95% CI (0.17, 2.28)^*^

* *p < 0.05*,

** *p < 0.01*,

*** *p < 0.001*.

*SA, suicide attempts; SI, suicide ideation; NSSI, non-suicidal self-injury*.

Mediation models focused on the indirect effects of IT on depression, well-being and psychological distress, respectively (36, 41, 43). It was reported that IT significantly mediated the association between gender identity and depressive symptomology (36). One study could not find a direct significant association but reported that perceived general stress mediated the relationship between IT and depression (41). Additionally, indirect associations between psychological distress and IT via rumination were reported (43). Interestingly, a mediation analysis of components of IT (shame and alienation) revealed that shame served as mediator between transnegative discrimination and depressive symptomology, but not alienation (48).

Three studies reported direct significant positive associations between IT and anxiety symptomology (17, 37, 43). Further, one study reported positive associations for both shame, alienation and anxious symptomology (48). Also, indirect associations between IT and anxiety were found (43). It was reported that IT was indirectly positively related to anxiety symptomology via rumination (43). Additionally, alienation and shame both served as mediator between transnegative discrimination and anxious symptomology (48).

### Relationships Between IT, NSSI, and Suicidal Tendency

In most of the reviewed studies IT is positively associated with SI (42, 44) and SA (38, 39). Further also 39 reported, that suicide risk including SI and SA is positively associated with IT. Only one study reported an opposite association for SI (45) (Table 8). However, the only study that examined the connection between IT and NSSI did not show a positive association (44).

Examining indirect effects of IT, one article found an indirect effect of IT on suicide risk (i.e., lifetime suicide ideation and/or suicide attempt, frequency of suicide ideation over the past 12 months, current threat of suicide attempt, and self-reported likelihood of suicidal behavior in the future) via depression in their sample of 329 self-identified trans individuals (40). They found perceived burdensomeness and thwarted belongingness to be meditators for the positive relation between IT and SI. However, no significant indirect effects of IT on NSSI were found (44).

### Relationships Between Protective Factors, Depression, and Anxiety

Four studies provide significant evidence that positive affect toward community is negatively associated with depression (17, 36, 46, 47) (Table 9). However, in the study of Jäggi et al. (47), this result was no longer significant in the mediation analysis. In one study pride toward the own trans identity was identified as another resilience factor for depression (17), but this association was not supported by another study (47). One study found community connectedness and pride combined to be protective factors for anxiety, however, the sample size was with *N* = 83 small (46). These findings are supported by Testa et al. (17) which found in a sample of 844 that more community connectedness and pride were related to less anxiety symptomology.

**Table 9:** Results overview, protective factors.

**Study**	**Outcome**	**Protective factors**	**Relationships of internalized transnegativity**	
			**Direct effects**	**Indirect effects**
Fredriksen-Goldsen et al. (36)	Depressive symptomology	a) Social support b) Positive feeling of community belonging	a) b = −2.96^***^ b) b = −1.29^***^	a) Path coefficient = 0.026^**^ b) Path coefficient = 0.006^*^
Testa et al. (17)	1) Depressive symptomology 2) Anxiety symptomology	a) Community connectedness b) Pride	1a) b = −0.21^*^ 1b) b = −0.17^*^ 2a) b = −0.19^*^ 2b) b = −0.20^*^	
Tebbe and Moradi (40)	Depressive symptomology	a) Friend support	a) b = −0.48^**^	b = −0.29, 95% CI (−0.132, −0.046)^**^
Brennan et al. (46)	1) Depressive symptomology 2) Anxiety symptoms 3) Lifetime SA	a) Older age b) Community connectedness and pride	1a) b = −2.712^*^ 2a) b = −3.993^**^ 2b) b = −0.058^*^ 3b) b = −0.281^*^	
Jäggi et al. (47)	Depressive symptomology	a) Community connectedness b) Pride	a) r = −0.22^**^ b) r = −0.13	(a) + (b): β = −0.67
Scandurra et al. (48)	Depressive symptomology Anxiety symptomology	a) General resilience	a) Shame r = −0.42^***^ b) Balienation r = −0.26^***^	a) r = −0.36^***^ b) r = −0.08

* *p < 0.05*,

** *p < 0.01*,

*** *p < 0.001*.

*SA, suicide attempts*.

Two studies reported indirect effects: one showed that IT significantly mediated the association between depression and anxiety (36). However, this was not supported by another reviewed study (47).

Further, general protective factors for depression have been reported. It was shown that social support (36), friend support (40), and general coping mechanisms defined as personal characteristics buffering the negative effects of stress and promoting adjustment (48) were both directly and indirectly negatively correlated with depression. However, general coping mechanisms resilience were only directly negatively associated with anxiety symptomology and not indirectly (48).

### Relationships Between Protective Factors, NSSI, and Suicidal Tendency

In one sample, the sum of pride and community connectedness was a protective factor against SA (46). No indirect effects or general protective factors for NSSI and suicidal tendency were reported.

## Discussion

This systematic literature review is the first examining the relationship between internalized transnegativity (IT) and mental health impairments according to the Gender Minority Stress Model (GMSM). The results from the reviewed literature suggest a positive association between IT and depression, anxiety, NSSI as well as suicidal tendency. Further, community connectedness and pride are identified as protective factor, which is negatively associated with mental health impairments. Despite the small number of studies and unsolved operationalization and measurement issues, these findings are especially important in order to support trans people in protecting themselves from negative psychological consequences of stigmatization and need to be taken into consideration in transition counseling and in the planification of psychotherapeutic interventions.

### IT, Depression, and Anxiety: Cognitive and Emotional Paths

The results of this review determined that IT was positively associated with depression, and anxiety in trans populations in most of the reviewed studies. Interestingly, in one sample, older trans people reported fewer depressive symptoms compared to younger trans people (36). One hypothesized explanation for this could be, that there are generational differences in IT and its relationship with psychological distress in feminine trans persons. As Jackman et al. (51) discussed, older trans feminine persons may have developed across their lifespan more stable coping skills and resilience factors and tend to be healthier in general compared to younger trans feminine persons. Another possible reason could be biographical, i.e., they are relieved to be finally an active part of the trans community after they repressed this desire for such a long time.

However, the reviewed literature lacks a conclusion on how exactly the internalization of negative beliefs and behaviors operates. On one hand, some mediation models emphasized the importance of cognitive-depressive processes such as rumination in the manifestation of depressive and anxiety symptoms in trans persons (43). The connection between involuntary and persistent focusing on the (trans-)negative events experienced and the development of depressive disorders has even been observed in a recent longitudinal study including young trans women (52). On this data basis, a connection of the GMSM to established theories like the response styles theory (53, 54) seems possible. This would open up the possibility to adapt evidence-based interventions for the reduction of rumination thoughts and consequently for the treatment of anxiety and depressive symptoms to the needs of trans persons. However, the results regarding the effects of IT on the development of anxiety symptomology remain less interpretable due to the small number of studies. Particularly in this area, further research is needed to confirm the accuracy of the correlations identified.

On the other hand, some studies highlighted the link between emotional processing mechanisms and the development of affective disorders (17). For example, Marshall et al. (39) were able to observe a correlation between experienced feelings of shame, guilt, low self-esteem and self-stigmatizing attitudes. This is also in line with Scandurra et al. (48), which reported a correlation between shame, alienation and mental health impairments. Due to their experience that the non-fulfillment of cis normative gender role stereotypes serves as the basis for their stigmatization, some trans people seem to devalue themselves by adopting this transnegative outside view and withdrawing socially due to a lack of (cis) passing. In fact, meta-analytic data show that external shame, which involves negative views of self as seen through the eyes of others, is associated with larger effect sizes to depression than internal shame, which involves negative views of self as seen through one's own eyes (55). These results suggest that shame not only should figure more prominently in understandings of the emotional underpinnings of anxiety and depressive symptoms, but also that reflection on shame mechanisms should play a more important role in the counseling and psychotherapeutic treatment of trans people.

Concurrently, other authors postulate that stigma-related stress creates elevations in general emotion dysregulation, social/interpersonal problems, and cognitive processes conferring risk for psychopathology (56). However, this hypothesis has been formulated for sexual and not for gender identity minorities. In view of the fact that these two groups are subject to similar, but not identical, exclusion mechanisms, comprehensive studies would be required to apply this hypothesis to the trans population.

### IT, NSSI, and Suicidal Tendency: Supportive Contacts Matter

Considering the extremely high prevalence of suicidal tendency among trans individuals (8, 11), it is of high relevance to understand the mechanisms behind the suicidal tendency in this population. With one exception, there was a positive relationship between suicidal tendency and IT in all the studies examined, which indicates the danger of internalized transnegative messages. This is in line with previous results from a systematic review (32). However, hardly any results were found on NSSI, which makes it difficult to conclude on the relationship between NSSI and IT.

Some preliminary data suggest that stigmatization is not only related to SI through experiences of internalized transphobia and negative expectations, but also that IT is associated with SI through interpersonal mediators as perceived burdensomeness and thwarted belongingness (42). In this way, the GMSM once again gains access to a general suicide theory (57). The pathways observed were specifically applicable for external stressors more related to social connections, including rejection and non-affirmation, reinforcing the idea that the daily life social isolation, which many trans persons experience, increases their sense of alienation. The establishment or maintenance of a supportive network of personal relationships is therefore crucial in order to be able to reduce IT and suicidality (58). However, further studies are needed to better elucidate the directionality of the found associations in a broader model.

### The Role of Protective Factors: Hardly Examined and Unclear

For the second research question regarding the GMSM's protective factors, results were not conclusive. Not all included studies could identify a significant protective effect of pride and community connectedness on depression and anxiety, NSSI, suicidal tendency (40, 43, 46). However, in the reviewed literature community connectedness showed to be the strongest protective factor for mental health impairments. These results suggest that trans individuals should be supported in connecting with other trans persons. This might help to reduce their depressive and anxiety symptomology and suicidal tendency. In some cases, the proximity to the trans community can even have an additional positive effect, since it can provide important support in the decision-making-process concerning the initiation of medical and/or social transition steps. However, this result should also be interpreted with care. Especially in cases in which trans persons can only count on social support if they completely hide their transitional biography (for example by living stealth, i.e., fitting into cisnormative gender role stereotypes and being viewed as clearly male or female), the proximity to the trans community could lead to the exacerbation of (anticipatory) anxiety symptoms. Accordingly, future measurements of “community connectedness” would have to take this dual role better into account. The protective influence of pride on depression, anxiety, NSSI and suicidal tendency is even less clear, because of the small number of studies investigating this factor included in this review. Thus, there is no doubt that there is an urgent need for research in this field.

### The GMSM: A Good Model With Measurement Issues

In addition to this quantitative problem, it must be mentioned, that some concepts proposed in the GMSM are also fraught with some inaccuracies, which makes it difficult to accurately measure the various dimensions and interpret the data. For example, the outness concept, which originally comes from LGB research (59), was adopted in the GMSM, although there are clear differences between pride mechanisms between the LGB and the trans population. In the case of gender identity, where different dimensions (non-binary vs. binary, feminine vs. masculine, etc…) intersect, the “outness question” is much more complex than the decision to present oneself as “trans” or not. Accordingly, “pride” has completely different implications for the trans masculine binary person with a cis-normative passing than for the woman who, against her will, is permanently read as “trans” by others. Not to mention the outing difficulties non-binary persons encounter when they come out and insist on being treated in the desired gender-neutral way. Therefore, the concept of outness might not fit exactly to all gender identity minorities which could explain the poor research results on this topic.

Another difficulty in the interpretation of the results is the strong connection of IT to other proximal minority stressors and to protective factors. Various studies have found dependencies between IT and community connectedness (17, 46, 47) or between concealment and pride (40). The last example shows how one and the same action can be counted in different dimensions of GMSM depending on the social context: while in unsafe environments, non-disclosure can be seen as protective factor, in safe environments, concealment can be described as proximal stress factor. Moreover, by mixing emotional, cognitive, and behavioral aspects of IT, newer concepts (21) do not help clarify this issue. Thus, more investigations derived from a consolidated theoretical framework that in contrast to the GMSM takes contextual factors into account are highly needed.

### Limitations

Several limitations have to be considered when interpreting the results of this review. The studies' samples consist mostly of Caucasian, western populations (USA, UK, IT, and Switzerland) and only English and German articles were included. Hence, the generalizability of these results is reduced and research that includes heterogeneous, multicultural samples is needed. In addition, all studies had a cross-sectional design, making it impossible to establish causal relationships between the factors studied. Studies with a longitudinal design would be helpful to overcome this problem. A main limitation is the inconsistent use of instruments to measure the various GMSM dimensions. Many of the applied measures in the selected studies were not tailored to the specific model definitions or adapted from other scales. Moreover, only few studies gave a detailed description or explanation regarding their modifications. Hence, the comparability of different scales and concepts of IT remains questionable. It would therefore be desirable for future studies to use instruments (i.e., “Gender Minority Stress and Resilience Measure” (17), “Transgender Identity Survey” (21) that relate to the GMSM and operate with its category definitions.

Another limitation is the focus of this research, which only included studies that could provide a clear definition of the identity labels used. We made this decision in order to examine a more homogenous group and with the intention of finding more robust data. Unfortunately, this step led to an underrepresentation of “non-binary” participants, as these identity labels were not or only very vaguely defined in some studies. It would therefore be important that future studies use better or more transparent definitions of terms to better understand the situation of this chronically underserved group.

Also, the recruitment strategy of most reviewed articles (17, 37–40, 42, 43) only via community groups is problematic. Trans persons who are not involved in the community might show higher IT leading to more mental health impairments. In this case, the investigated sample would represent an a priori healthier group compared to the average trans population. Therefore, future studies should work with a more heterogenous sample. In addition, it would also be important to investigate possible differences between gender identity groups regarding IT and its relationship with depression, anxiety, NSSI and suicidal tendency, since they might experience different forms of discrimination.

### Clinical Implications

Professionals advising and treating trans people should be informed about the existence of the gender minority stress and its implications with mental health impairments. Therefore, trans people with anxiety or depressive symptoms should be asked about discriminatory experiences and potential internalized stress factors with sufficient care. In cases where there are clear indications for an existing IT, a combination of cognitively-oriented (e.g., education) and emotionally-oriented (e.g., strengthening self-acceptance) elements could lead to a reduction of this harmful factor. Further it is crucial to strengthen the social support system consisting of significant others outside and/or especially also within the trans community. In sum, with the reduction of minority stressors and promotion of resilience factors, there is hope that trans individuals can reduce existing mental problems in order to achieve a better quality of life.

### Conclusion

The results of the reviewed studies indicate that self-stigmatization plays an important role in the development of depression, anxiety and suicidal tendency in trans people. This first available data indicates links between self-stigmatization and other general mental health stressors such as rumination and thwarted belongingness. Self-stigmatization thus has both a direct and indirect effect on these symptoms and needs to be taken into consideration in transition counseling and in the planification of psychotherapeutic interventions. It could be targeted and treated using existing therapeutic approaches. Community connectedness showed to be the strongest protective factor for mental health impairments, otherwise the findings regarding protective factors were less conclusive.

Therefore, more research is needed to better understand the phenomenon of IT itself and to test specific treatment approaches. It is also important to further develop the GMSM, in particular, the context-dependency of some central terms should be better defined.

## Data Availability Statement

The original contributions presented in the study are included in the article/Supplementary Material, further inquiries can be directed to the corresponding author/s.

## Author Contributions

MI wrote the manuscript together with DG. The systematic research was performed by KS and MI. The methodological and writing supervision was done by AS, DG, and JG. All authors have approved the manuscript and the order of authorship.

## Conflict of Interest

The authors declare that the research was conducted in the absence of any commercial or financial relationships that could be construed as a potential conflict of interest.

## Footnotes

^1^Trans persons are people who do not or not entirely identify with the sex assigned to them at birth. This well accepted term was proposed by Lev (1) and includes for example trans, transgender, female-to-male, transsexual.

^2^Sexual minorities are defined as group whose sexual identity, orientation or practices differ from the majority of the surrounding society. Oftentimes, they are also refered to as LGB (5).
