# Supplementary material for: Relationship of Internalized Transnegativity and Protective Factors With Depression, Anxiety, Non-suicidal Self-Injury and Suicidal Tendency in Trans Populations: A Systematic Review
Source: Front Psychiatry. 2021 May 20;12:636513. doi: 10.3389/fpsyt.2021.636513 (PMC8172993; doi:10.3389/fpsyt.2021.636513)
Supplement: Supplementary file 1 [file Data_Sheet_1.docx]

Appendix

| Table 1 |  |
| --- | --- |
| *Search terms Pubmed* |  |
| P(ATIENTS) | trans* OR transsex* OR transgender OR gender dysphor* OR gender minorit* OR LGBT OR gender identit* OR male-to-female OR female-to-male OR transmen OR transwomen |
| I(NTERVENTIONS) | transphobia OR stigma* OR minority stress OR sexual-stigma OR sexual stigma OR transnegativ* OR cissexism OR discrimination |
| C(OMPARISON) | internalized transphobia OR internalised transphobia OR internalized stigma OR internalised stigma OR self-stigma OR self stigma OR internalized cissexism OR internalised cissexism OR internalized transnegativ* OR internalised transnegativ* |
| O(OUTCOME) | suicide risk OR suicide attempts OR suicide OR suicidal ideation OR suicide ideation OR suicidal behaviour OR suicidality OR self-harm behaviour OR self-harm OR non-suicidal self-harm OR self-injury behaviour OR self-injury OR non-suicidal self-injury OR “suicide, attempted” OR “suicide attempted/psychology” |

| Table 2 |  |
| --- | --- |
| *Search terms Web of Science* |  |
| P(ATIENTS) | trans* OR transsex* OR transgender OR gender dysphor* OR gender minorit* OR LGBT OR gender identit* OR male-to-female OR female-to-male OR transmen OR transwomen |
| I(NTERVENTIONS) | transphobia OR stigma* OR minority stress OR sexual?stigma OR transnegativ* OR cissexism OR discrimination |
| C(OMPARISON) | internali?ed transphobia OR internali?ed stigma OR self?stigma OR internali?ed cissexism OR internali?ed transnegativ* |
| O(OUTCOME) | suicide risk OR suicide attempts OR suicide OR suicidal ideation OR suicide ideation OR suicidal behaviour OR suicidality OR self-harm behaviour OR self-harm OR non?suicidal self-harm OR self-injury behaviour OR self-injury OR non?suicidal self-injury |

| Table 3 |  |
| --- | --- |
| *Search terms Psycinfo, Embase, CINAHL* |  |
| P(ATIENTS) | trans* OR transsex* OR transgender OR gender dysphor* OR gender minorit* OR LGBT OR gender identit* OR male-to-female OR female-to-male OR transmen OR transwomen |
| I(NTERVENTIONS) | transphobia OR stigma* OR minority stress OR sexual-stigma OR sexual stigma OR transnegativ* OR cissexism OR discrimination |
| C(OMPARISON) | internalized transphobia OR internalised transphobia OR internalized stigma OR internalised stigma OR self-stigma OR self stigma OR internalized cissexism OR internalised cissexism OR internalized transnegativ* OR internalised transnegativ* |
| O(OUTCOME) | suicide risk OR suicide attempts OR suicide OR suicidal ideation OR suicide ideation OR suicidal behaviour OR suicidality OR self-harm behaviour OR self-harm OR non-suicidal self-harm OR self-injury behaviour OR self-injury OR non-suicidal self-injury |
